# Prehospital Cervical Spine (C-spine) Stabilization and Airway Management in a Trauma Patient: A Review

**DOI:** 10.7759/cureus.54815

**Published:** 2024-02-24

**Authors:** Utkarsh M Waghmare, Akhilesh Singh

**Affiliations:** 1 Accident and Trauma Care Technology, Jawaharlal Nehru Medical College, Datta Meghe Institute of Higher Education and Research, Wardha, IND; 2 Emergency Medicine, Jawaharlal Nehru Medical College, Datta Meghe Institute of Higher Education and Research, Wardha, IND

**Keywords:** endotracheal intubation, airway management, cervical spine injury, prehospital management, cervical cord

## Abstract

Severe traumatic damage to the brain-to-body signaling bundle that results in bruising and a partial or total tear of the spinal cord is known as a spinal cord injury (SCI). SCI may develop at the time of an event or after. It can also develop while handling the patient and can worsen during the transportation of the patient. So prehospital care is crucial to prevent or minimize SCI. Prehospital care involves examining the patient, immobilizing the cervical spine (C-spine), providing cardiovascular support (keeping the mean arterial blood pressure over 85 mmHg), and carefully managing the airway (possibly intubating the patient using manual in-line stabilization (MILS)). Methylprednisolone (MPS) and other pharmacological treatments have not been shown to offer clinically meaningful and essential benefits for people with SCI. The therapeutic use of MPS in patients with SCI in the prehospital context is no longer supported. Additionally, whether or not pharmaceutical drugs will be effective in therapeutic hypothermia is unknown. When performing endotracheal intubation on these patients, the potential for C-spine damage is always considered. During intubation, the MILS approach significantly reduces C-spine movement. The MILS method, however, can potentially restrict mouth opening and result in subpar laryngoscopic vision. These issues can be handled using the recently developed video laryngoscope, such as Airtraq laryngoscope and AirWay Scope (AWS). Compared to a direct laryngoscope, the AWS and Airtraq laryngoscope reduced the improvement of intubation conditions and the acceleration of tracheal intubation through the occiput-C1 and C2-C4 levels of the C-spine extension movement.

## Introduction and background

The annual incidence of spinal cord injuries (SCI) in the United States is estimated to be 43-77 per million people, or around 20,000 cases annually. Twenty percent of these individuals pass away before hospital admission. According to one study, about 200,000 people in the United States had this prevalence of SCI. The ratio of men to women was 4:1. Around four billion dollars was predicted to be spent annually in the United States on SCI therapy in 1990 [1]. Damage to the cervical spine (C-spine) is a critical factor in deciding between life and death. Hypercapnia, aspiration pneumonia, and respiratory arrest can cause subsequent hypoxic brain damage and endotracheal intubation problems [2]. One of the most crucial components of prehospital emergency treatment is airway management, which is essential for a patient's short-term survival and potential for recovery. Among the three methods that emergency medical services (EMS) commonly use, the gold standard for managing airways is generally acknowledged to be endotracheal intubation (ETI) over supraglottic airway devices (SGA) and intubation. Success rates for emergency techniques vary significantly due to various factors, despite being regulated for high complications and prehospital airway treatment situations [3-7]. For emergency response systems (ERS), spinal immobilization in the civilian sector has established protocols. Regarding combat casualties who might suffer spinal cord or spinal column injuries, there is a clinical practice guideline in place [8]. The first step in managing SCI is recognising the injury. A spinal column injury that is not correctly diagnosed or treated can cause a permanent loss in a patient's ability to function, a neurologic deficit, and in some cases, even death [9]. An acutely injured patient must be treated cautiously before being brought to the hospital. In the case of severe closed-head trauma, the possibility of cervical SCI is higher, occurring in about 2% of all blunt trauma patients [10]. Patients with acute SCI run the risk of neurologic decline [11]. A source of potential future damage is accidental spinal cord manipulation in the presence of an unstable spinal column [12].

## Review

Search methodology

**Figure 1:**
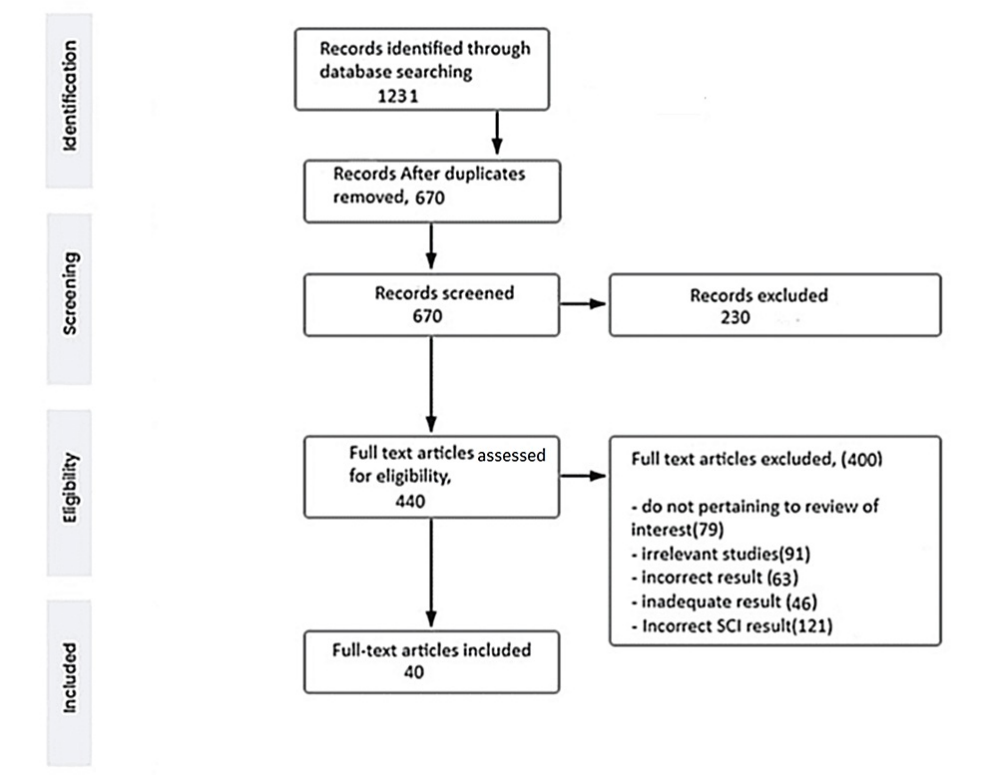
PRISMA flow chart of search strategy PRISMA: Preferred Reporting Items for Systemic Reviews and Meta-Analyses; SCI: Spinal cord injury Own creation. Source [celegence]

We undertook a systematic search through PubMed, Google Scholar, and Cochrane Central Register of Controlled Trials (CENTRAL) in June 2022 using keywords such as "C-spine stabilization", "cervical cord", "prehospital management", "cervical spine injury", "airway management", and "endotracheal intubation" (("cervical cord" [Title/Abstract]) OR ("cervical cord" [MeSH Terms]), (“prehospital management” [Title/Abstract])) OR ("prehospital management" [MeSH Terms]), ("airway management" [Title/Abstract]) OR ("airway management" [MeSH Terms]) AND ("endotracheal intubation" [Title/Abstract]) OR ("endotracheal intubation” [MeSH Terms])). The authors independently reviewed papers based on title and abstract, ensuring inclusion criteria were met before proceeding to full texts, with study selection based on specific criteria (Figure 1): (1) C-spine stabilization, (2) airway management, (3) C-spine injury, (4) prehospital management, and (5) English language. The following were the exclusion criteria: (1) airway suctioning, (2) case study, (3) surveys, (4) animal studies, (5) non-English language research, and (6) not an empirical study (e.g., theory or opinion articles).

Prehospital spine immobilization and the battlefield

Like civilian trauma systems, prehospital care for war emergency departments with a suspicious spinal column or spinal cord injury follows treatment philosophies focused on conforming to the Advanced Trauma Life Support (ATLS) protocol [13]. Performance in difficult, dangerous, and harsh situations is the most crucial distinction between war casualty care and trauma treatment in domestic populations. While in conflict, casualties are evacuated by moving backward through the care echelons, further away from the combat area, and with more advanced medical supplies. The first person to respond on the scene, providing care in the most hazardous setting, provides case 1 care. The first responder is usually another military member, a corpsman with limited resources or a combat medic with minimal medical knowledge to undertake life-saving operations to control bleeding and manage an airway and essential life support. Instead of maintaining spinal stability, the first responder's priority is quickly removing the injured comrade from harm. Spine immobilization has no place on the battlefield since the lives of combat casualties and combat medics must be preserved. As a result, spine immobilization is typically delayed on the battlefield during extrication until circumstances permit, and minimum battlefield intervention of the polytrauma combat victim is advocated. When moving the patient, the patient's head needs to be neutrally positioned and aligned with the body's axis if spine immobilization is required during extrication. The patient shouldn't be coerced into a position that causes them unnecessary pain or a deformity that differs from their natural posture [14,15]. The use of prehospital management that might postpone the need for surgical operations for patients with potentially survivable injuries is also being contested by a growing body of evidence [16-18].

Prehospital selective spine immobilization

Prehospital spine immobilization is required for combat casualties who meet the subsequent clinical requirements that raise suspicion of a spine or spinal cord injury: having spine discomfort or tenderness, having a focused neurologic deficit, having a changed level of awareness, having a suspected fractured extremity, or having other distracting severe injuries. In a 6500-patient multicenter prospective civilian study on trauma, Domeier et al. discovered that the previously mentioned parameters were typically predictive of patients who might have sustained C-spine injuries and require C-spine immobilization [19-22]. Prehospital C-spine immobilization should be taken into account for the damage that causes momentary loss of consciousness or amnesia after a significant blast or explosive injury, after a fall from a height, after being ejected or falling from a bike, bus, etc., or following a rollover injury [23]. Spinal instability does not show in many trauma cases but spinal immobilization will always be beneficial to prevent neurological damage to the spinal cord if suspecting SPI without spinal instability. So spinal immobilization must always be done as a part of prehospital treatment of trauma patients till SPI is ruled out by radiological investigation [24]. There are hazards associated with observational studies, and spinal immobilization has discovered that inflexible collars can result in iatrogenic pain [25].

Trauma airway management goals

The objectives of appropriate oxygenation and ventilation should be the main emphasis of trauma airway treatment. EMS doctors should quickly reassess the situation to decide whether to escalate from fundamental to advanced airway treatments. Effective airway management is essential for critically injured patients, and procedures should be focused on preventing hypotension, hypoxemia, and hypercarbia [26], especially for those suffering from burns, hemorrhagic shock, and traumatic brain injury (TBI). In the care of trauma patients, training usually focuses on managing complex airway techniques, yet this approach ignores the greater priority of maintaining oxygenation and ventilation. The two underlying ideas are: (1) ventilation and oxygenation should be the primary goals of rescue care rather than specialized airway procedures, and (2) basic techniques should be used to treat airway problems, and only when necessary should advanced therapies be used. There is a need to emphasize the significance of ventilation and oxygenation [27].

Patient with trauma: drug-assisted airway management 

Some trauma patients, particularly those who have suffered a TBI, may not have intact protective airway reflexes, which is why airway management by endotracheal intubation is necessary to maintain airway patency and to protect from aspiration of gastric content for which pharmacological support (drugs) is used to manage the airway smoothly and without fail. The phrase drug-assisted airway management (DAAM) refers to several techniques that use pharmacological aids to promote smooth and successful advanced airway insertion [28]. In trauma patients, especially those with hemorrhagic shock and TBI, DAAM medicines may deteriorate patient physiology. In some patients, hemorrhagic shock may change the necessity for medication administration, necessitating the use of lower doses [29]. Trauma patients could be physiologically unstable, necessitating ongoing observation while the airway is managed. This is especially true for TBI patients as DAAM may worsen hypotension and hypoxia due to their longer duration of action, thus negatively impacting patient outcomes [30,31].

Transport immobilization techniques and devices

It is important to always keep the combat casualty safe to avoid further harm, regardless of injury, because ground vehicles or helicopters employed for extrication are frequently uncovered in challenging geographic terrain and weather. Spinal immobilization is carried out when the necessary instruments and resources are available for constructing a war emergency room for the evacuation of combat casualties from the front lines to a case 2 or 3 medical institution with a suspected spinal cord or spinal column injury. To hold the patient in place over the thorax, forehead, and extremities, the American College of Surgeons recommends utilizing lateral support devices, a rigid backboard, straps, a semirigid cervical collar, or tape [32,33]. Because of their great lateral motion, the authors recommend avoiding transferring war casualties with spine or spinal cord injuries using the usual log roll approach [34,35]. Adopting the high arm in endangered spine technique is recommended, in which the doctor or corpsman kneels on the opposite side of the patient with both lower extremities flexed and the near arm over the patient's chest. As another medical professional or corpsman maintains in-line head and neck stability, the victim is gently rolled and scooped to the side, where a backboard or transfer device is placed [36].

Spinal injury protocol (level 3) for combat support hospital

The first course of care for a combat casualty with polytrauma should comprise a primary survey using the stepwise ATLS technique upon arrival at a case 3 medical facility. Correctly identifying and treating spinal column and spinal cord injury are prioritized in losses with hemodynamic instability. Preventing further SPI from hypotension, hypoxia, dependent edema, hyperthermia, and hypothermia should be the primary goal of initial care during resuscitation of a patient with suspected spine injury. The secondary damage cascade of SCI is the best opportunity to reduce the impact of subsequent neurologic deterioration. Utilizing the American Spinal Injury Association's guidelines, every attempt should be made to record a neurologic exam that is as detailed as feasible, including motor testing, dermatomal examinations, lumbar and sacral root evaluations, sensory testing, and exam of the rectum. To avoid pressure sores, patients must be taken off the backboard within two hours after its insertion. While inspecting the neck, back, and buttocks as part of the secondary survey, the backboard should consistently be taken away. Recognition of the injury is the first step in managing a combat casualty with spinal cord and column damage. The C-spine precautions can be lifted from a war casualty who is conscious and free of neck pain or soreness. This does not require further radiological analysis [37,38]. Computed tomography (CT) has replaced plain radiography as the primary screening method for trauma patients in the last 10 years. A spiral CT traumagram is recommended for battle casualties who are unconscious or unable to perform an accurate clinical examination due to changed mental status or distracting injuries. Compared to plain radiographs, 99 percent of all thoracic spinal fractures may be found with a CT scan of the spine and cervical column, and it offers a more precise assessment of skeletal anomalies and spinal canal impairment [39]. Odontoid, anteroposterior, and lateral views of the C-spine X-ray should make up the first imaging sequence in the absence of a CT scan. Training usually focuses on complex airway techniques in the care of trauma patients for maintaining oxygenation and ventilation. A case 4 medical treatment center has the first magnetic resonance imaging (MRI) capability for locating hidden fractures or ligamentous spine injuries. Dynamic flexion and extension C-spine radiographs are not advised by the authors to be taken in the operating room; instead, they should wait until two weeks following the accident, when the pain and muscle spasms have adequately reduced. Due to the paucity of equipment in the theatre and the inconsistent capacity to accurately measure reduction and placement, the usefulness of halo immobilization in the combat theatre scenario is constrained and not practical. Despite its patient holding capacity and critical care capabilities, a case 3 medical treatment facility's main goal is quickly getting the battle casualty ready for aeromedical transport to a permanent treatment facility. After being medically stabilized, the battle casualty is transported from the scene of the incident to a case 4 or 5 medical care center. The interdisciplinary trauma team must exercise increased caution during the aeromedical procedure to identify the characteristics and safety of spinal injuries. The proper identification and safety of war casualties with spinal cord injury or spinal column trauma leads to the prevention of neurologic deterioration during aeromedical evacuation [40].

Table 1 describes the characteristics of the studies included in the review.

**Table 1:** List of some studies referred to for this article with some important findings drawn from the studies AAOS: American Academy of Orthopaedic Surgeons; HAINES: High Arm IN Endangered Spine

Author	Year	Country	Findings
Bernhard M et al. [1]	2005	Germany	Since 25% of spinal cord injury (SCI) may develop or be worsened after the initial occurrence, prehospital care of SCI is crucial
Smith JP et al. [17]	1985	California	Transport time to the hospital was shorter than the IV establishment time
Domeier RM et al. [20]	1999	USA	AAOS recommendations emphasize spinal cord immobilization based on symptoms and physical findings of potential spinal injury
Domeier RM et al. [21]	1999	USA	Injury mechanism doesn't impact spinal injury prediction in this population
Kang DG et al. [23]	2011	USA	Discusses protocol for treating combat casualties with suspected spinal cord injury, including considerations for medical evacuation and aeromedical transport
Chan D et al. [25]	1994	California	Spinal immobilization can cause pain in healthy persons
Kim MW et al. [26]	2018	South Korea	Hypoxia significantly increases mortality and disability in hypotensive individuals
Jarvis JL et al. [28]	2022	USA	For advanced airway placement in patients with respiratory failure, changed mental status, or airway compromise, emergency medical services (EMS) use drug-assisted airway management (DAAM)
Egan ED et al. [29]	2020	USA	Hemorrhagic shock physiology changes intravenous anesthetics' pharmacokinetics and pharmacodynamics, affecting dose-concentration relationships
Swain A et al. [35]	1990	USA	The spinal cord is prone to damage in the cervical region and near the thoracolumbar junction due to narrower spinal canals and vertebral displacement
Gunn BD et al. [36]	1995	Australia	Position unconscious persons in HAINES modified recovery position for a neck injury, reducing the risk of spinal cord damage

## Conclusions

The varied spectrum of C-spine injuries encompasses a range of complexities and implications for patient care and management. From the relatively common whiplash injuries to the more severe fractures and dislocations, each type presents unique challenges in diagnosis, treatment, and rehabilitation. Understanding the distinct characteristics, mechanisms, and associated risks of different C-spine injuries is crucial for healthcare professionals to deliver timely and appropriate interventions, thereby minimizing long-term complications and optimizing patient outcomes. Moreover, ongoing research and advancements in diagnostic modalities, surgical techniques, and rehabilitation protocols continue to enhance our ability to effectively address these injuries and improve patient prognosis. Ultimately, a comprehensive approach that integrates clinical expertise, evidence-based practices, and patient-centered care is essential in navigating the complexities of C-spine injuries and promoting optimal recovery and quality of life for affected individuals. The prevention of negative effects during flight depends on the preparation for aeromedical transfer. In order to help people with SPI adapt and achieve independence and well-being, a multidisciplinary strategy comprising medical professionals, rehabilitation specialists, and support networks is necessary. It is important to correctly identify the battle casualty who has suffered a spinal column or spinal cord damage, stabilize their medical condition, and immobilize them for aeromedical evacuation to a treatment or nearest health center capable of proper investigation and treatment. The results of this review are in-depth and thorough and can guide future research, policy, practice, and education to enhance prehospital airway care and breathing assistance and improve patient outcomes.
